# Maturation of the long-latency auditory ERP: step function changes at start and end of adolescence

**DOI:** 10.1111/j.1467-7687.2007.00619.x

**Published:** 2007-09

**Authors:** Dorothy VM Bishop, Mervyn Hardiman, Ruth Uwer, Waldemar von Suchodoletz

**Affiliations:** 1 Department of Experimental Psychology, University of Oxford UK; 2 Department of Child and Adolescent Psychiatry and Psychotherapy, University of Munich Germany

## Abstract

The auditory event-related potential (ERP) is obtained by averaging electrical impulses recorded from the scalp in response to repeated stimuli. Previous work has shown large differences between children, adolescents and adults in the late auditory ERP, raising the possibility that analysis of waveform shape might be useful as an index of brain maturity. We reanalysed auditory ERPs from samples previously described by Albrecht, von Suchodoletz and Uwer (2000) and Uwer, Albrecht and von Suchodoletz (2002), using the intraclass correlation (ICC) as a global measure of similarity of an individual's waveform to a grand average comparison waveform for each age band. Three developmental periods were clearly distinguished: 5 to 12 years, 13 to 16 years, and adulthood. However, within each of these periods, there was no evidence of any developmental progression with age.

## Introduction

**Figure 1:**
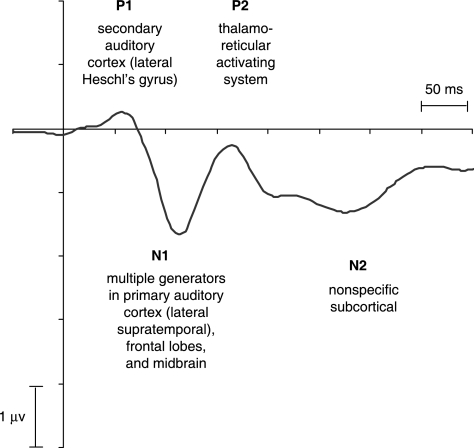
Grand average auditory ERP from 21 adults studied by Albrecht et al. (2000). For each listener, the ERP was obtained by averaging responses to over 900 sinusoidal 1000 Hz tones, 175 ms in duration. Summary information about the generators for each peak is based on Velasco, Velasco and Velasco (1989), Näätänen (1992) and Ponton et al. (2000).

The long-latency auditory event-related potential (ERP) is a waveform that emerges when averaging electrical impulses recorded from the scalp in response to repeated stimuli. Figure 1 shows the typical waveform from an adult that is obtained from a central frontal electrode (Fz) when responses are averaged from multiple presentations of a 175 ms tone to which no response is required. The distinctive positive and negative peaks that occur 50 ms or later after stimulus onset are labelled by polarity (P = positive and N = negative) and order of occurrence. Figure 1 summarizes what is known about the generators for each peak. Electrode site is not an accurate reflection of the origin of ERP deflections; because auditory stimulation activates more than one pathway from ear to brain, most peaks seen in the long-latency auditory ERP represent summed neural activity from several distinct generators. For instance, frontally recorded N1 is thought to reflect the combined activity of as many as six generators, including a major source in auditory cortex.

**Figure 2:**
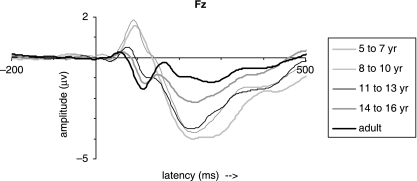
Grand average auditory ERPs for tones groups by age group at electrode Fz.

The long-latency auditory ERP holds promise as an index of the brain's responses to auditory stimuli in real time while minimizing performance demands on the listener. This makes it particularly suitable for studying auditory processing in children, whose ability to attend to psychoacoustic tasks may be too limited to allow accurate measurement. However, the interpretation of auditory ERPs in children is complicated by the fact that auditory areas of the brain continue to develop into the teens (Moore, 2002) and this is reflected in changes in the long-latency auditory ERP (Eggermont & Ponton, 2003; Wunderlich & Cone-Wesson, 2006). Figure 2 shows sample grand average waveforms for different age bands from 5 years to adulthood from the current study.

Although such developmental changes have been documented in several studies, we are aware of no study to date that has considered how variation *within* an age band compares to variation *between* age bands. One way of thinking about this question is to ask how accurately one might be able to predict a child's age from knowledge of the auditory ERP. If the variation within an age band is large relative to variation between age bands, then this prediction will be poor, even if developmental trends are evident in the grand averages. There are several reasons why it is important to establish how tight the relationship is between age and auditory ERP. First, if one is using auditory ERPs in a study comparing a clinical and control group, one needs to know whether it is reasonable to group together children of different ages. If age exerts a substantial influence on the ERP, then this may mask effects of interest when comparing two groups. It is not uncommon to find clinical studies that group together children spanning an age range of several years. To date, decisions on which age bands to use have been made in a largely ad hoc fashion. One goal of this paper is to establish guidelines for setting age bands based on empirical data. A second reason for studying this issue is to discover whether the auditory ERP could be useful as an index of brain maturity. The notion of immature neurological development is sometimes raised as a possible etiology of children's language and learning difficulties, but to date the evidence has been largely based on behavioural measures (Bishop & Edmundson, 1987; Wright & Zecker, 2004). If we find much greater variability between age groups than within age groups, then the auditory ERP could be used as an index of brain maturation. A further reason for investigating changes in the auditory ERP with age is to gain insight into underlying changes in brain structure and function that may be responsible for developmental trends.

Before we can investigate such questions, we have to consider how to characterize the auditory ERP at different ages. The usual approach involves measuring the latency and amplitude of peaks and troughs in the waveform, such as P1, N1, P2 and N2. As documented by Johnstone, Barry, Anderson and Coyle (1996) and Ponton, Eggermont, Kwong and Don (2000), these do show age-related changes. However, as shown in Figure 2, the N1 and P2 peaks are not always seen in ERPs of children under 12 years of age (Albrecht *et al*., 2000; Ponton *et al*., 2000), although the likelihood of observing these components in children increases with stimulus onset asynchrony (SOA; Paetau, Ahonen, Salonen & Sams, 1995; Ceponiené, Cheour & Näätänen, 1998; Ceponiené, Rinne & Näätänen, 2002; Gilley, Sharma, Dorman & Martin, 2005). When a peak is not present in a waveform, we should beware of assuming that the generator is inactive; Ponton *et al*. (2000) argued that N1 was generated in children, but could be masked by phase cancellation from a large P1. Source localization studies indicate that generators for late AEPs are in the same locations for adults and children, but have different weightings at different ages (Albrecht *et al*., 2000; Ceponiené*et al*., 2002; Ponton, Eggermont, Khosla, Kwong & Don, 2002), so that in children the waveform looks different from that of adults.

Given that measurement of latency and amplitude of N1 and P2 peaks is not always feasible in pre-adolescent children, and that these peaks in any case may not be a good guide to underlying component structure (Luck, 2005), if we want to study early cortical responses to sounds in children we need a method that does not depend on identifying specific peaks. McArthur and Bishop (2004) addressed this problem using the intraclass correlation (ICC). They first computed a normative grand average waveform for typically developing children in a given age band, and then computed the intraclass correlation (ICC) between that grand average and an individual child's waveform in the same region. The ICC is similar to the more familiar Pearson correlation coefficient, except that it is sensitive to absolute size of the values in two arrays. Thus whereas the Pearson correlation coefficient would be unchanged by adding a constant to all the values in one of two correlated arrays, the ICC would decrease as the mean of the two arrays became more discrepant. The ICC thus gives a global index of similarity between two waveforms; a low ICC can arise if there are either amplitude or latency differences in peaks and troughs: McArthur and Bishop (2004) showed that the ICC agrees well with ratings of waveform similarity made by untrained observers on the basis of visual inspection. Using this method, McArthur and Bishop (2004) and Bishop and McArthur (2005) showed that teenagers with specific language impairment (SLI) tended to have waveforms that were more similar to those of younger controls than to their own age group. However, these studies were limited by small sample size; the normative waveforms were derived from only 16 typically developing children divided into two broad age bands (above and below 14 years).

Another limitation of the previous studies by Bishop and colleagues was that the ICC analysis was based on a restricted set of electrodes at fronto-central sites, where the auditory ERP tends to be maximal. Previous developmental studies have proposed that there are age-related changes in the pattern of activation seen in the auditory ERP, as well as in the amplitude and latency of peaks. In particular, children tend to show relatively greater peak amplitudes than adults at temporal sites (Bruneau & Gomot, 1998).

The aim of the current study was to consider two related questions: first, how does auditory ERP variation *within* an age group compare with the variation *between* age groups? Second, how far does the ICC provide a sensitive measure of maturational level of the auditory ERP in typically developing children? Bishop and McArthur (2005) looked at typically developing children in two broad age bands: 10–14.5 years and 14.5–19 years, and found clear differences in their late auditory ERPs. The question arises as to whether, with a larger sample, and using a larger array of electrodes, we would be able to make finer discriminations between children of different ages. We had available for analysis data from standard nonspeech (tone) and speech (monosyllable) stimuli, so were also able to consider whether ERPs to these two stimulus types showed a similar developmental course.

## Methods

### Participants

The data came from the sample of 108 individuals aged from 5 to 30 years studied by Albrecht *et al*. (2000). The age distribution is shown in Table 1. Individuals with psychiatric disorders, peripheral hearing loss or neurological impairment were excluded from the sample.

**Table 1:** Numbers of females and males in sample, by age

	Female	Male	Total
5 to 6 years	3	8	11
7 to 8 years	8	12	20
9 to 10 years	9	7	16
11 to 12 years	7	6	13
13 to 14 years	13	1	14
15 to 16 years	6	7	13
20 to 30 years	6	15	21

### Electrophysiological recording procedure

ERPs were recorded as participants were passively presented with either tones or syllables played in four blocks each of 333 stimuli with constant stimulus-onset asynchrony of 1 second. An oddball paradigm was used in which a standard stimulus was presented on 70% of trials and one of two deviants on the remaining trials; however, the analyses reported here are concerned solely with responses to standard stimuli. The standard tone stimulus was a 1000 Hz tone (rise and fall time 10 ms) and the speech stimulus was a digitized syllable /da/ spoken by a female German speaker. Both were of 175 ms duration. Stimuli were presented to the right ear via earphones at 86 dB SPL. During the recording children watched a silent videotape and were instructed to ignore the tones.

Data were acquired using a Neuroscan system with sampling rate of 256 Hz, with recordings from silver silver-chloride electrodes positioned according to the International 10/20 system at 22 sites: FP1, FP2, F3, F4, F7, F8, FZ, FT9, FT10, T3, T4, T5, T6, C3, C4, CZ, P3, P4, PZ, O1, O2, OZ. Electrodes were referenced to the right mastoid, and horizontal (HEOG) and vertical (VEOG) eye movements were recorded. An online bandpass filter was set with limits at 0.1 and 30 Hz. Automatic artefact rejection was applied offline to exclude all epochs with voltage exceeding ± 80 µv. Offline the recordings were re-referenced to the average of all electrodes (except for HEOG and VEOG) and a spline fit was used to convert to a sampling rate of 250 Hz: this latter step was taken to give compatibility with future data from studies currently under way in the first author's lab.

### Computation of the intraclass correlation

There are several approaches to computation of the ICC, depending on the purpose of the analysis. The method we adopt is equivalent to the SPSS one-way random ICC, which is different from the ICC transform provided with Neuroscan Scan 4.3 software. The ICC was computed between corresponding datapoints of two waveforms: a *normative* waveform, which was the grand average for a group of children in the same age range, and a *comparison* waveform, which is the waveform of an individual child. The comparison waveform was never included in the average used for the normative waveform: i.e. when computing the ICC for a typically developing child, the normative grand average was based on all the other children of the same age. Because the datapoints in a waveform are not independent of one another, one cannot use conventional tables of statistical significance to evaluate the probability of obtaining a given ICC by chance. Bishop and McArthur (2005) randomly generated simulated waveforms with levels of autocorrelation similar to those seen in ERPs to establish probabilities of obtaining ICCs of different magnitudes. When computing means and conducting statistical analyses that assume normality, the Fisher transform was first applied, but all data reported here were transformed back to ICCs.

## Results

### Topographic maps

**Figure 3:**
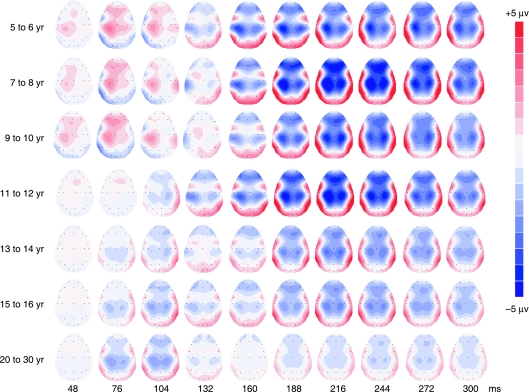
Topographic maps showing amplitudes of ERP at all electrodes for tone stimuli over the interval 48 to 328 ms post-onset.

**Figure 4:**
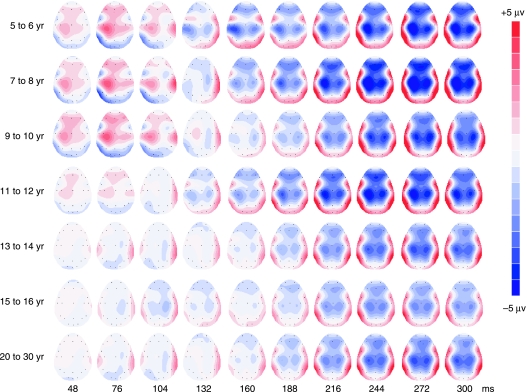
Topographic maps showing amplitudes of ERP at all electrodes for syllable stimuli over the interval 48 to 328 ms post-onset.

Before conducting the ICC analysis, topographic maps were created to visualize developmental changes in the distribution of auditory ERP activity with age. These were created as cartoons using Scan 4.3 software using default options for global interpolation. Figures 3 and 4 show topographic maps for different age bands for the time period from 48 to 328 ms post stimulus onset.

These data may be compared to those of Bruneau and Gomot (1998), who presented topographic maps for eight children aged 7 to 9 years, eight aged 10 to 12 years and eight adults. Their stimuli were 750 Hz tone bursts of 200 ms duration, delivered monaurally at random to left or right ears at intensity of 70 dB SPL, with random ISI of 1 to 2 s. A nose reference was used. Note that our procedure differed from that of Bruneau and Gomot in terms of SOA (constant at 1 s), stimulus frequency (1000 Hz), stimulus duration (175 ms), ear of presentation (right ear), intensity (86 dB SPL), and referencing (average of all electrodes). Despite these differences, we can see many of the features described by Bruneau and Gomot in the map in Figure 3, as follows:

1. N1b, a fronto-central negativity around 100 ms post-onset of stimulus, is not apparent in children aged younger than 10 years, but can be seen in the older children.
2. N1b is accompanied by positive potentials at temporal sites.
3. The topographic distribution of negativity in the fronto-central regions around 100 ms becomes broader with increasing age.
4. A fronto-central negative wave is evident in children, peaking around 250 ms (N2). A similar negativity was seen in adults, but it is of smaller amplitude and is topographically more focal.
5. Negativity is seen at T3 (left temporal site) in children but not in adults in the time period from around 70 to 180 ms. It is noteworthy that a similar left-lateralized bias was observed by Bruneau and Gomot, even though their stimuli were presented at random to left and right ears, suggesting that the laterality of the activity is not a consequence of the right ear presentation that we used.

Bruneau and Gomot focused principally on the N1 wave, and they did not comment on the P1, which precedes it. In Figure 3 one can see striking developmental changes in this component, with marked fronto-central positivity, especially on the left side, around 70 ms for children below 11 years of age.

Figure 4 shows comparable brain maps elicited by the syllable /da/. Note that although the speech and tone stimuli were matched for intensity and duration, they were not matched for acoustic content or complexity, and so it would be premature to conclude that any differences in waveforms were due to the speechlike nature of the syllabic stimuli. In many respects, the topographic maps resemble those seen for tones, with the following exceptions:

1. In children aged below 11 years, P1 is larger and starts earlier than for tones.
2. N1 is seen around 100 ms in those aged 11 years and over, but it is smaller than for tones.
3. Positivity at temporal sites around 100 ms is more lateralized to the right than for tones, and is also more extended in time.

One notable feature of the topographic maps for both tones and speech stimuli is that the patterns of activation are strikingly different for younger children and adults, but the change does not appear to be gradual. On visual inspection, it is hard to see any developmental trends from 5 to 10 years. A change appears around 11 to 12 years, and, in the tone data, the impression is of a smooth developmental trend after 12 years toward earlier and larger N1b, and smaller and more focal N2.

### Intraclass correlation analysis for tones at different electrode sites

Grand average waveforms in each age band are shown for tone stimuli in Figure 2 for electrode FZ, which was the electrode that gave the largest auditory ERP. (To improve readability of the graph, children are here divided into age bands spanning 3 years, but analyses were conducted on 2-year bands, as shown in Table 1.)

ICCs with normative age-appropriate grand means were computed across two intervals: (a) 0 ms to 552 ms (139 data points), incorporating P1, N1, P2 and N2; (b) 100 to 228 ms (33 data points), which is the same as that used by Bishop and McArthur (2005), and encompasses N1 and P2. The normative waveforms were based on the age ranges shown in Table 1.

### Sensitivity of ICCs to developmental level in typically developing children

**Figure 5:**
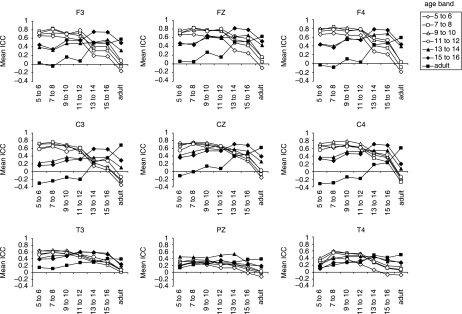
Mean ICC over 0 to 552 ms in each age band, relative to normative waveforms from different age bands (x-axis) at nine electrodes. Simulations of comparable data by Bishop and McArthur (2005) gave estimates of .50, .69 and .72 as values corresponding to the 5%, 2.5% and 1% levels of significance (one-tailed).

If the ICC is sensitive to developmental level, we should expect the mean ICC for typically developing children to be highest when compared with the normative waveform of their own age group, and to fall off when compared with normative waveforms from other age groups. The mean ICCs for tone stimuli at nine electrode sites over the interval 0 to 552 ms are shown for each age band in Figure 5. There are several striking features of these data. First, differences in ICCs between age groups are evident for frontal and central electrodes, but are only small for the temporal electrodes and PZ, where the mean ICCs, though positive, often fail to reach the 5% significance level, even with the same-age reference group. For frontal and central electrodes, ICCs are mostly significant with the same-age reference group, but there is no suggestion of linear developmental change: if there had been, we would have expected the ICCs for each age group to peak when compared with their own age normative waveform. Instead, the data suggest three broad groups. For children aged from 5 to 12 years, high ICCs are found with all four normative waveforms in that age band, suggesting that there is little variation in the late auditory ERP either within or between age for children in this age range. For those aged 13 to 14 years, the ICCs are generally rather low when compared with all age groups, whereas the 15- to 16-year-olds have a peak in the function with both their own age band and the next youngest. For the 20- to 30-year-olds, the mean ICC is close to zero with normative waveforms for the youngest children, low for the normative waveform for teenagers, but mostly significant with their own normative age group.

**Figure 6:**
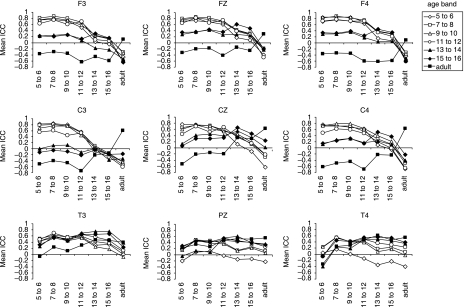
Mean ICC over 100 to 228 ms for typically developing children in each age band, relative to normative waveforms from different age bands (x-axis) at nine electrodes. Simulations of comparable data by Bishop and McArthur (2005) gave estimates of .48, .53 and .66 as values corresponding to the 5%, 2.5% and 1% levels of significance (one-tailed).

Figure 6 depicts an equivalent analysis conducted on the ICCs computed across the time window of 100 to 228 ms. The data at frontal and central sites again show a clustering of plots for three main age bands: children aged from 5 to 12 years, teenagers aged 13 to 16 years, and young adults. At frontal and central sites, the adult ICCs with child waveforms tend to be negative, probably reflecting the fact that a waveform containing N1 and P2 will be going up at the time point when a child's waveform will show a continuous decrease in amplitude. It is noteworthy also that the average ICC is low and non-significant for the adult group with their own normative waveform: this is an indication that the grand average is not representative of individuals in the group: this could arise, for instance, if one were averaging waveforms in which the timing of peaks and troughs differed from one individual to another.

The plots in Figures 5 and 6 show the mean ICCs, but if one wants to use the ICC to assess auditory maturity in an individual, a key question is how much variation there is around this mean – i.e. how common is it for a typically developing child to have an ICC more similar to that of an older or younger age group? Tables 2 and 3 provide data pertinent to this question, for the 0–552 ms and 100–228 ms intervals, respectively, for electrode C3. To create these tables, for each case we identified the age band with which each participant had the highest ICC. This is the ‘maximal ICC group’ and can be thought of as a measure of ‘auditory ERP age’. Electrode C3 was selected because it gave the strongest association between a person's chronological age and the maximal ICC group, although values were in a similar range for all fronto-central electrodes. The association was significant for both intervals; for 0 to 552 ms, χ^2^= 170.6, d.f. = 36, *p* < .001; for 100–228 ms, χ^2^= 130.7, d.f. = 36, *p* < .001. For both intervals, 49% of cases had ICCs that best matched their own age group. The percentages whose maximum ICC fell either in their own age group or in an adjacent age group was 82% for the 0 to 552 ms interval and 77% for the 100–228 ms interval. Table 2 again suggests three broad age groups: children under 10 years of age virtually never had a waveform that resembled that of a child over this age, and it was rare for the 11- to 12-year-olds to resemble teenagers. Those aged 13 to 14 years showed a wider spread of maximal ICCs, with some resembling one of the teenage or adult groups, and the remainder resembling younger cases. Fifteen- to 16-year-olds never resembled a child under 10 years. Adults usually had a maximal ICC with the adult normative group. Overall, these data suggest that the broad distinction between children, adolescents and adults is well indexed by the ICC, but there is little differentiation within each of these age bands.

**Table 2:** Numbers of cases in each age band with maximum ICC at each normative age band, time window 0 to 552 ms, electrode C3: those with maximum ICC at own age shown in bold

	Normative group giving maximum ICC							
	
Age band	5 to 6	7 to 8	9 to 10	11 to 12	13 to 14	15 to 16	20 to 30	Total
5 to 6	**3**	5	3	0	0	0	0	11
7 to 8	3	**11**	6	0	0	0	0	20
9 to 10	0	4	**11**	0	1	0	0	16
11 to 12	1	6	1	**3**	1	1	0	13
13 to 14	0	0	3	4	**3**	2	2	14
15 to 16	0	0	0	2	6	**3**	2	13
20 to 30	0	0	0	0	1	1	**19**	21

**Table 3:** Numbers of cases in each age band with maximum ICC at each normative age band, time window 100 to 228 ms, electrode C3: those with maximum ICC at own age shown in bold

	Normative group giving maximum ICC							
	
Age band	5 to 6	7 to 8	9 to 10	11 to 12	13 to 14	15 to 16	20 to 30	Total
5 to 6	**3**	4	4	0	0	0	0	11
7 to 8	5	**11**	4	0	0	0	0	20
9 to 10	2	4	**7**	2	0	0	1	16
11 to 12	2	4	2	**4**	0	1	0	13
13 to 14	0	1	4	3	**4**	0	2	14
15 to 16	4	1	0	1	3	**1**	3	13
20 to 30	0	0	0	0	2	0	**19**	21

Overall a similar picture emerges for Table 3, which restricts analysis to the N1-P2 region, except that it was very rare to find any participant, teenager or otherwise, whose waveform in the N1-P2 region resembled that of 15- to 16-year-olds. Furthermore, several children in this age band had a maximum ICC with a much younger age band. This suggests that over this interval adolescents have very variable waveforms and the grand average is not representative of individuals constituting this age group.

Parallel analyses were conducted with ERPs elicited by syllables, and are summarized in Table 4. This shows the average ICC with own-age normative group for each age: the higher this value, the less variability in waveforms within an age band. Table 4 also shows the percentages of cases with maximal ICC group either matching their age group, or falling in an adjacent age band; this gives an indication of how age-specific the waveform is at a given electrode. The general pattern of results at frontal and central sites was comparable for that seen with tones, but there was more inter-individual variability within age bands, and hence a trend for lower ICCs overall. Also, the ICCs with the age-appropriate normative group tended to be very low at parietal and temporal electrodes, seldom reaching significance for any age band, especially when attention was restricted to the 100–228 ms interval. The highest levels of agreement between actual age and maximal ICC group were seen at CZ; over the 0–552 ms interval, 43% of cases had perfect agreement, falling to 41% for the 100–228 ms interval.

**Table 4:** Mean ICCs with own age band by age, and overall % cases with maximal ICC at own age band or within one age band: results from speech stimulus over interval of 0–522 ms and 100–228 ms

	Mean ICC with own age band comparison group							% cases with max ICC	
		
Electrode	5–6	7–8	9–10	11–12	13–14	15–16	20–30	Exactage band	Within 1age band
0–552 ms									
F3	.729	.793	.588	.787	.594	.542	.304	39.8	75.0
FZ	.729	.843	.678	.775	.727	.704	.443	43.5	76.9
F4	.724	.812	.685	.741	.621	.641	.283	39.8	75.0
C3	.715	.748	.639	.598	.560	.351	.425	41.7	75.9
CZ	.780	.777	.738	.689	.683	.642	.463	42.6	78.7
C4	.756	.770	.755	.640	.642	.566	.324	42.6	77.8
PZ	.185	.430	.345	.060	.394	.247	.315	28.7	60.2
T3	.520	.417	.487	.353	.494	.495	.217	32.4	62.0
T4	.107	.266	.339	.301	.513	.125	.389	29.6	66.7
100–228 ms									
F3	.663	.766	.384	.645	.229	.000	.003	35.2	68.5
FZ	.591	.782	.431	.615	.434	.143	.028	33.3	65.7
F4	.659	.779	.472	.617	.223	−.067	−.188	31.5	63.0
C3	.581	.640	.422	.347	.194	−.305	.470	38.9	72.2
CZ	.471	.527	.336	.454	.249	.077	.418	40.7	68.5
C4	.607	.644	.425	.508	−.053	−.123	.376	34.3	68.5
PZ	.071	−.053	−.111	−.232	−.119	.024	.488	17.6	44.4
T3	.373	.135	.003	.420	.144	−.162	−.169	17.6	43.5
T4	.450	.182	.120	−.074	−.098	−.394	.160	27.8	51.9

## Discussion

Using a sample of participants aged from 5 years to adulthood, we used the ICC statistic to compare each individual's waveform with that of a normative grand average. Results suggested that auditory ERPs could be classified into three broad age bands, covering the age ranges 5 to 12 years, 13 to 16 years, and adulthood. The ICC did not seem useful as an index of ‘auditory ERP age’ within each age band, but was effective at discriminating between these three broad age groupings.

One methodological implication of these results is that if one studies a sample spanning a wide age range from childhood to late adolescence, there will be significant age-associated variation that will mask group differences. In the current sample, there was a fairly sharp change in ERP waveform at Fz around 12 years of age, when N1-P2 started to emerge. This is reminiscent of results by Sharma, Kraus, McGee and Nicol (1997) for long-latency ERPs elicited by speech stimuli. Others have observed N1-P2 at somewhat earlier ages, possibly related to use of different stimuli and methods (e.g. Paetau *et al*., 1995; Tonnquist-Uhlén, Borg & Spens, 1995; Ponton *et al*., 2002; Gilley *et al*., 2005), but in general, one does not reliably see N1-P2 below 10 years of age unless one uses very slow presentation rates. An exception is a study by Martin, Barajas, Fernandez and Torres (1988), who identified N1-P2 in 12/17 6- to 7-year-olds and 17/18 9- to 10-year-olds. It is possible that their use of very brief (24 ms) tones, and/or an active paradigm, where children counted deviant responses, accounts for this difference (see also Jirsa & Clontz, 1990). Overall, it seems unwise to group pre- and post-adolescents together when conducting studies comparing clinical and control groups.

A complementary conclusion is that the auditory ERP is not as useful as had been hoped for testing ‘maturational lag’ accounts of developmental disorders. Bishop and McArthur (2005) suggested that evidence on this issue could be obtained by seeing whether a child with a developmental disorder had an auditory ERP that resembled that of a younger typically developing child. In their study, they found evidence of this, when comparing adolescents with SLI with younger control children. However, the current data suggest that they had been fortunate in studying a specific age range where the auditory ERP does show dramatic developmental change. In pre-adolescent children, comparisons of ERPs with those of younger children would not throw light on the ‘maturational lag’ hypothesis, because the auditory ERP does not show developmental change between the ages of 5 and 12 years. Nevertheless, there might be some value in extending this approach to younger children, as we know that auditory ERPs do show substantial change between infancy and young childhood (Pasman, Rotteveel, Maassen & Visco, 1999; Wunderlich & Cone-Wesson, 2006).

Step-like rather than continuous change is unusual in development. Even if a sudden transition occurs in individuals, one would not expect to see a sharp discontinuity in group averaged data, unless the timing of the transition was fairly uniform from one individual to another. Otherwise, each age group would contain a proportion of children who had and had not made the transition, with the proportion increasing with age, to give the impression of a gradual development. One might wonder whether the step function observed here was affected by sampling error: i.e. if by chance we included rather more immature pre-teens and more mature teenagers, an uncharacteristically sharp transition might be seen. Unfortunately, there are few developmental ERP studies with sufficient data to provide a comparison, but it is of interest to note that the broad age groupings that we found here are similar to those seen in the data of Ponton *et al*. (2000) for P1 amplitude. They found that the age plots for the lateral electrodes C3 and C4 showed abrupt changes between children aged from 5 to 10 years, 11 to 15 years, and 16 years and over. Their plots for N1 and P2 did not show this discontinuity, but this could be an artefact, because these authors had to exclude data from numerous young children who did not have these peaks.

What neurophysiological changes might underlie such abrupt developmental shifts? The timing of the transitions suggests that we need to consider changes in auditory neurophysiology that coincide with the onset and offset of puberty (see also Oades, Dittmann-Balcar & Zerbin, 1997). The nature of these changes remains unclear, though several possibilities have been proposed, including axonal growth, increased myelination, and synaptic pruning (Illing, 2004). Ponton *et al*. (2000) suggested that stepwise developmental changes might reflect a sudden decrease in the synaptic density of auditory cortex. However, longitudinal MRI data from Giedd *et al*. (Giedd, Blumenthal, Jeffries, Castellanos, Liu, Zijdenbos, Paus, Evans & Rapoport, 1999) and Gogtay *et al*. (Gogtay, Giedd, Lusk, Hayashi, Greenstein, Vaituzis, Nugent, Herman, Clasen, Toga, Rapoport & Thompson, 2004) indicated that grey matter in frontal and parietal lobes peaked at around 11 to 12 years and then declined, whereas temporal lobe grey matter continued to increase gradually throughout childhood and adolescence, only starting to level off and decline in adulthood. In addition, the dorsolateral prefrontal cortex only started to lose grey matter at the end of adolescence. At the more microscopic level, Amunts, Schleicher, Ditterich and Zilles (2003) have shown that area 44 in the inferior frontal gyrus (part of classical Broca's area) attains an adult-like, left-larger-than-right asymmetry around 11 years of age. Thus the dramatic changes in the late auditory ERP observed around the start of adolescence may reflect synaptic pruning affecting regions of the frontal lobe rather than auditory cortex (see also Bruneau, Roux, Guerin, & Barthélémy, 1997). The later changes seen in young adults could relate either to changes affecting auditory areas in the temporal lobes, or to maturation of the frontal lobes continuing beyond adolescence (Amunts *et al*., 2003).
